# Approach to the management of paediatric HIV spontaneous controllers

**DOI:** 10.4102/sajid.v37i1.399

**Published:** 2022-06-30

**Authors:** Peter Zuidewind, Mark Cotton, Shaun Barnabas, Anita Janse Van Rensburg, Gert van Zyl, Carli Gordijn

**Affiliations:** 1 Department of Paediatrics and Child Health, Faculty of Medicine and Health Sciences, University of Stellenbosch, Cape Town, South Africa; 2 Department of Virology, Faculty of Medicine and Health Sciences, University of Stellenbosch, Cape Town, South Africa

**Keywords:** HIV, spontaneous controllers, paediatrics, HIV functional cure, infectious diseases

## Abstract

Paediatric HIV spontaneous controllers (HSCs) are a unique and understudied population with potential to inform alternative treatment options for patients living with HIV. As HSCs are so rare and often not recognised prior to antiretroviral treatment (ART) initiation, it can be difficult for clinicians to optimally manage this group. We describe the diagnosis, history and management of three paediatric HSCs, two girls and a boy who were followed for 2, 1.25 and 10.4 years, respectively, before starting ART. All had low but detectable viral loads throughout follow-up but mostly marginally low CD4:CD8 ratios. The reason for starting ART in all was a gradual tendency to poorer virological control. This case series should assist in recognising paediatric HSCs. Clinical dilemmas arising in the management of paediatric HSCs include arriving at a correct HIV-positive diagnosis, correct diagnosis as an HSC, as well as whether to initiate ART. Decision-making for initiation of ART in paediatric HSCs should be individualised. Factors supporting ART initiation in these patients included increased frequency of viral load blips, increasing detectable viral load, CD4 percentage and CD4:CD8 ratio. Other factors included Hepatitis C serology and highly sensitive C-reactive protein. All three patients ultimately required ART, which supports universal initiation of ART in paediatric HSCs, but further research is required.

## Introduction

Human immunodeficiency virus (HIV) controllers are a unique and rare group of individuals living with HIV who can control HIV replication without combination antiretroviral therapy (ART). The term ‘HIV controllers’ is an ambiguous term and refers both to those who have never taken ART (usually labelled controllers) and those receiving time-limited ART shortly after primary infection (often referred to as post-treatment controllers). We propose the term HIV spontaneous controllers (HSCs) to describe those who can control HIV without ART exposure. Whilst most HSCs are adults, a small number of children have been thus described.^1,2,3^ HIV spontaneous controllers can be broadly categorised as either elite or viraemic controllers. Elite controllers maintain an undetectable viral load in the absence of ART, whilst viraemic controllers maintain low but detectable viral loads (usually < 1000 HIV-1 ribonucleic acid (RNA) copies/mm^3^) in the absence of ART.^4,5,6^ A useful definition is to have at least three low or undetectable viral loads over more than a year.^4^ Transient elevated viral load ‘blips’ are generally acceptable as long as the next viral load measurement returns to the previous trend.^7^ In this case series, viral loads < 1000 HIV-1 RNA copies/mm^3^ were used to define the previous trend.

Whilst ART is the mainstay of current HIV management, it does not come without side effects and drug interactions.^8,9^ Therefore, having additional treatment options available is vital.

Understanding how adult HSCs suppress viraemia may hold the key to discovering new and safer methods of controlling HIV replication, such as informing which HIV epitopes to include in a therapeutic vaccine.^10^ This same principle applies for paediatric HSCs, who may be immunologically different from adult HSCs.^11^ Paediatric HSCs do also present important clinical dilemmas that are sometimes difficult to answer. These include misdiagnosis as HIV-negative,^12^ undisclosed administration of ART mimicking an HSC and whether to start ART.^13^ This case series presents three paediatric HSCs who were followed-up at a research unit in Cape Town, South Africa between 2013 and 2020 (see Table 1). Patients 1 and 2 were referred to participate in ART studies, whilst Patient 3 was identified as an HSC at a peripheral clinic and referred to the site for inclusion in this case series.

**TABLE 1:** Patient demographics and duration of control.

	Ethnicity	Gender	Maternal PMTCT	Age at presentation	Age at loss of control	Duration of documented control
Patient 1	Mixed race, father of African descent	Female	None reported	2 years 9 months	3 years 11 months	14 months of elite control
Patient 2	African	Male	None reported	12 years 2 months	13 years and 6 months	16 months of viraemic control
Patient 3	African	Female	None reported	13 years 9 months	20 years 10 months	7 years 1 month of elite control

PMTCT, prevention of mother-to-child transmission.

## Case presentations

### Patient 1

#### Presentation

This girl presented at the age of two years nine months. She is mixed race and has a father of African descent. HIV was diagnosed at the age of two years eight months and she was referred for participation in an ART study. Her mother reports her own HIV diagnosis at approximately three months gestation (three years prior to the patient’s diagnosis) and reports not taking ART either as prevention of mother-to-child transmission (PMTCT) or for herself. Neither information on the nature of the mother’s diagnosis nor any clinic records to confirm the lack of ART were available. The mother reported that the child tested HIV-negative after birth, but there was no record of this. The infant received nevirapine for one month as PMTCT. The mother commenced tenofovir, emtricitabine and efavirenz in February 2013, when her CD4 count was 209 cells/mm^3^ (23.5%). Her adherence was suboptimal, often missing doses on weekends. Her earliest viral load was 100 HIV-1 RNA copies/mm^3^ after four months of ART. Patient 1 was exclusively breastfed until six months of age, which continued to a variable degree until four years of age. As the child’s HIV diagnosis was made at two years of age, antivirals in breastmilk may have contributed to the suppressed viral load at diagnosis. Unfortunately, ART blood levels were not measured. The child’s acquisition of HIV was presumed to be perinatal as she was diagnosed relatively young. The mother had HIV, no blood products were given and there was no suspicion in the family or by the researchers of sexual abuse of the child.

#### Investigations

Human immunodeficiency virus diagnosis was through a 4th generation single HIV Ag/Ab combination assay (HIV Combo; Abbott Diagnostics, Abbott Park, IL) at two years nine months of age. At this time her CD4 count was 1817 cells/mm^3^ (35.20%). An HIV DNA PCR (COBAS^®^ AmpliPrep/COBAS^®^ TaqMan^®^, HIV-1 Qualitative Test, v2.0; Roche, Manheim, Germany) was indeterminate, cycle threshold (Ct) > 33 and relative fluorescence increase (RFI) < 5. At the same time point, the plasma viral load (Abbott *m2000sp;* Des Plaines, IL, US) was 132 HIV-1 RNA copies/mm^3^. A series of confirmatory tests, namely HIV antibody test (Alere Determine; Matsuhidai, China), HIV Unigold FDA test (Uni-Gold™ Recombigen^®^ HIV-1/2; Jamestown, New York, NY, US) and HIV Western blot test (GS HIV-1 Western; Redmond, Washington, DC, US) were all positive.

#### Follow-up

A repeat viral load a month later in April 2013 was < 40 HIV-1 RNA copies/mm^3^. Subsequently, three monthly viral loads over 14 months suggested elite control (see Table 2a and b)^14^. Her CD4:CD8 ratio was depressed; however, no other cause for this was detected. In May 2014, the viral load increased significantly to 58 978 HIV-1 RNA copies/mm^3^, roughly coinciding with cessation of breastfeeding. This calls the initial diagnosis of an HSC into question as ART drug levels were not performed. However, the viral load subsequently fell spontaneously to 228 HIV-1 RNA copies/mm^3^, supporting HSC status. Abacavir, lamivudine and efavirenz were started in February 2015, with a viral load of less than 40 HIV-1 RNA copies/mm^3^ within three months.

**TABLE 2a:** Laboratory data for patient 1.

Date	Age (years)	Viral load (HIV-1 RNA copies/mm^3^)	Log	CD4 abs (Cells/mm^3^)	CD4 %	CD8 abs(Cells/mm^3^)	CD8 %	CD4:CD8
21 February 2013	2			1817	35.20	-	-	-
05 March 2013		132	2.121	1713	32.08	1998	37.42	0.86
24 April 2013		< 40	< 1.600	1906	45.17	1395	33.06	1.37
08 August 2013	3	< 40	< 1.600			-	-	-
05 November 2013		< 40	< 1.600	1897	43.61	-	-	-
31 January 2014		< 40	< 1.600	-	-	-	-	-
02 May 2014		58 978	4.770	-	-	-	-	-
08 August 2014	4	228	2.360	1691	39.10	-	-	-
01 November 2014		1900	3.280	-	-	-	-	-
23 January 2015		3370	3.530	-	-	-	-	-
06 February 2015†				1729	42.00	1765	43.00	0.98
08 May 2015		< 40	< 1.600	1917	43.00	1785	40.00	1.08
30 October 2015	5	< 20	< 1.300	1574	41.00	1382	36.00	1.14
25 October 2016	6	< 20	< 1.300	-	-	-	-	-
17 October 2017	7	< 20	< 1.300	-	-	-	-	-
31 October 2018	8	< 100	< 2.000	-	-	-	-	-
28 October 2019	9	< 20	< 1.300	-	-	-	-	-

HIV, human immunodeficiency virus; ART, antiretroviral therapy; RNA, ribonucleic acid; abs, absolute.

† , ART started.

**TABLE 2b:** Laboratory reference ranges.

Age	CD4 Abs	CD4 %	CD8 Abs	CD8 %	CD4:CD8
2–5 years old	700–2200	28–47	490–1300	16–30	> 1.0
6–11 years old	650–1500	31–47	370–1100	18–35	> 1.0
12–17 years old	530–1300	31–52	330–920	18–35	> 1.0
Adult	332–1642	28–51	170–811	12–38	> 1.0

*Source:* Shearer WT, Rosenblatt HM, Gelman RS, et al. Pediatric AIDS Clinical Trials Group. Lymphocyte subsets in healthy children from birth through 18 years of age: The Pediatric AIDS Clinical Trials Group P1009 study. J Allergy Clin Immunol. 2003 Nov;112(5):973–80. https://doi.org/10.1016/j.jaci.2003.07.003

abs, absolute.

### Patient 2

#### Presentation

A 12 year old boy of African descent (on maternal and paternal sides) was referred to the research site for screening for an ART study. His mother’s HIV status was diagnosed during pregnancy at an unknown gestation, and she reported not receiving ART during pregnancy. Clinic records were not available for verification. The boy received nevirapine as PMTCT for 10 days after birth, which is not the standard of care, but the mother reports not being instructed appropriately on administration.

#### Investigations

According to the mother, his HIV was diagnosed at birth but ART was not started. Clinical records for verification were unavailable. There was no subsequent history of ART according to the mother or the child, with no other caregiver on ART in the household. Antiretroviral therapy drug levels were again not performed. When screened for an ART study in March 2017, his viral load was 62 HIV-1 RNA copies/mm^3^. His HIV status was then confirmed with a positive HIV1/2 Ab/Ag Elisa (HIV Combo; Abbott Diagnostics, Abbott Park, IL, US) and a positive HIV-1 RNA/DNA PCR (COBAS^®^ AmpliPrep/COBAS^®^ TaqMan^®^ HIV-1 Qualitative Test v2.0; Roche Molecular Systems, Inc., Branchburg, NJ, US), Ct ≤ 33 and RFI ≥ 5.

#### Follow-up

Three monthly viral loads documented 16 months of viraemic control. It is not known what the viral load trajectory was prior to the age of 12 years, and there may have been a previous undocumented period of elite control. His CD4:CD8 ratio was low with no other cause found. His viral load increased to 2352 HIV-1 RNA copies/mm^3^ in June 2018, requiring ART initiation (see Table 3a and b)^14^. He began abacavir, lamivudine and efavirenz in June 2018, and his viral load was less than 20 HIV-1 RNA copies/mm^3^ within three months.

**TABLE 3a:** Laboratory data for patient 2.

Date	Age (years)	Viral load (HIV-1 RNA copies/mm^3^)	Log	CD4 abs (Cells/mm^3^)	CD4 %	CD8 abs (Cells/mm^3^)	CD8 %	CD4:CD8
03 March 2017	12	62	1.79	585	32.51	-	-	-
23 August 2017		248	2.39	-	-	-	-	-
12 January 2018	13	298	2.47	356	27.0	568	43.0	0.63
12 April 2018		600	2.78	502	32.78	-	-	-
26 June 2018†		2352	3.37	552	30.0	754	41.0	0.73
27 September 2018		< 20	< 1.30	-	-	-	-	-
20 December 2018		-	-	714	42.0	578	34.0	1.24
23 July 2019	14	-	-	376	34.5	410	37.6	0.92

ART, antiretroviral therapy; RNA, ribonucleic acid; abs, absolute.

† , ART started.

**TABLE 3b:** Laboratory reference ranges.

Age	CD4 Abs	CD4 %	CD8 Abs	CD8 %	CD4:CD8
2–5 years old	700–2200	28–47	490–1300	16–30	> 1.0
6–11 years old	650–1500	31–47	370–1100	18–35	> 1.0
12–17 years old	530–1300	31–52	330–920	18–35	> 1.0
Adult	332–1642	28–51	170–811	12–38	> 1.0

*Source:* Shearer WT, Rosenblatt HM, Gelman RS, et al. Pediatric AIDS Clinical Trials Group. Lymphocyte subsets in healthy children from birth through 18 years of age: The Pediatric AIDS Clinical Trials Group P1009 study. J Allergy Clin Immunol. 2003 Nov;112(5):973–80. https://doi.org/10.1016/j.jaci.2003.07.003

abs, absolute.

### Patient 3

#### Presentation and initial diagnosis

This girl presented to health services at the age of six years when her mother died of an unknown HIV-related illness. Both parents were of African descent. No PMTCT information was available.

#### Investigations

Her HIV was diagnosed using an unknown test. She was not initiated on ART and her first viral load (< 40 HIV-1 RNA copies/mm^3^) was at the age of 13 years (see Table 4a and b)^14^. Her positive HIV status was then confirmed with a positive HIV Combo Ag/Ab assay (HIV Combo; Abbott Diagnostics, Abbott Park, IL, US) and a confirmatory positive HIV Antibody Elisa test (VIDAS HIV DUO Ultra; Biomérieux, Marcy-l’Etoile, France). Her viral load remained undetectable until she presented to the research site at 16 years old. Her positive status was confirmed again with a positive HIV Combo Ag/Ab assay (HIV Combo; Abbott Diagnostics, Abbott Park, IL, US) and a positive HIV qualitative PCR (COBAS^®^ AmpliPrep/COBAS^®^ TaqMan^®^ HIV-1 Qualitative Test v2.0; Roche Molecular Systems, Inc., Branchburg, NJ), Ct ≤ 33 and RFI ≥ 5.

**TABLE 4a:** Laboratory data for patient 3.

Date	Age (years)	Viral load (HIV-1 RNA copies/mm^3^)	Log	CD4 abs (Cells/mm^3^)	CD4 %	CD8 abs (Cells/mm^3^)	CD8 %	CD4:CD8
25 January 2010	10	-	-	712	33.60	-	-	-
29 July 2011	12	-	-	823	28.60	-	-	-
17 July 2012	13	-	-	772	30.60	-	-	-
05 February 2013		< 40	< 1.60	782	30.20	-	-	-
05 August 2013	14	< 40	< 1.60	862	31.42	-	-	-
10 February 2014		< 150	< 2.20	890	30.22	-	-	-
04 August 2014	15	< 150	< 2.20	698	28.35	-	-	-
02 February 2015		47	1.67	771	20.09	-	-	-
19 June 2015	16	< 40	< 1.60	607	20.00	1482	49.0	0.41
07 August 2015		< 20	< 1.30	-	-	-	-	-
13 January 2016		< 20	< 1.30	-	-	-	-	-
02 March 2016		< 20	< 1.30	783	28.59	-	-	-
24 June 2016	17	< 20	< 1.30	720	26.68	-	-	-
07 October 2016		43	1.63	778	25.65	-	-	-
10 January 2017		< 20	< 1.30	818	26.18	-	-	-
10 April 2017		< 20	< 1.30	614	25.22	-	-	-
27 September 2017	18	< 20	< 1.30	596	26.80	-	-	-
04 April 2018		< 20	< 1.30	-	-	-	-	-
29 August 2018	19	< 20	< 1.30	833	25.00	1765	53.0	0.47
12 December 2018		< 20	< 1.30	821	27.00	1581	52.0	0.52
29 March 2019		< 20	< 1.30	541	25.15	-	-	-
03 July 2019	20	56	1.75	713	23.60	1747	57.9	0.41
19 September 2019		< 20	< 1.30	432	23.43	-	-	-
19 December 2019		52	1.72	633	23.60	1585	59.0	0.40
19 March 2020		122	2.09	769	25.47	-	-	-
25 June 2020	21	< 20	< 1.30	640	21.40	1767	59.1	0.36
29 September 2020		< 100	< 2.00	-	-	-	-	-
11 December 2020		< 50	< 1.70	495	22.00	1375	61.1	0.36
04 June 2021†	22	< 20	< 1.30	509	18.20	-	-	-
29 September 2021		< 20	< 1.30	-	-	-	-	-
02 January 2022		< 50	< 1.70	-	-	-	-	-

ART, antiretroviral therapy; RNA, ribonucleic acid; abs, absolute.

† , ART started.

**TABLE 4b:** Laboratory reference ranges.

Age	CD4 Abs	CD4 %	CD8 Abs	CD8 %	CD4:CD8
2–5 years old	700–2200	28–47	490–1300	16–30	> 1.0
6–11 years old	650–1500	31–47	370–1100	18–35	> 1.0
12–17 years old	530–1300	31–52	330–920	18–35	> 1.0
Adult	332–1642	28–51	170–811	12–38	> 1.0

*Source:* Shearer WT, Rosenblatt HM, Gelman RS, et al. Pediatric AIDS Clinical Trials Group. Lymphocyte subsets in healthy children from birth through 18 years of age: The Pediatric AIDS Clinical Trials Group P1009 study. J Allergy Clin Immunol. 2003 Nov;112(5):973–80. https://doi.org/10.1016/j.jaci.2003.07.003

abs, absolute.

#### Follow-up

Her viral load remained undetectable for the following four years, except for viral blips, all followed by undetectable results. In 2019–2020, she had two consecutive detectable viral loads at 52 and 122 HIV-1 RNA copies/mm^3^, achieving elite control status for a total of seven years one month. ARV drug levels (efavirenz, lopinavir, ritonavir, atazanavir, darunavir and dolutegravir) were below the detectable limit, confirming her status as an HSC. Recurrent viral load blips, a persistently low CD4:CD8 ratio with no other detectable cause and a CD4 percentage lower than 40% favoured the initiation of ART. Factors that did not support this were her Hepatitis C serology, which was negative, and a highly sensitive C-reactive protein (CRP) test, which was 0.7 mg/L (will discussed further). She agreed to initiate ART after discussing these concerns. She commenced tenofovir, lamivudine and dolutegravir, and three months later her viral load was still < 20 HIV-1 RNA copies/mm^3^. No further CD4 or CD8 results were available after initiating ART.

## Discussion

Different terms have been used to categorise patients who suppress HIV viraemia or maintain CD4 counts in the absence of ART. As has been described above, we propose the term HSC to distinguish patients who spontaneously suppress viraemia and those that do so after prior ART (post-treatment controllers). Post-treatment control has been postulated by some to represent HSCs who were started on ART before virological testing,^15^ but the incidence of post-treatment control is higher than that in HSCs.^11^ Another difference is that HSCs have much higher levels of HIV-specific CD8+ T-cell responses than post-treatment controllers.^16^ As has been mentioned, HSCs can be divided into elite and viraemic controllers based on the level of viraemia, but another group of patients similar to viraemic controllers called transient aviraemics (TAs) have also been described.^1^ These patients have undetectable viral loads on less than three occasions in one year, not meeting the criteria of elite control.^1^ Another term used before widespread accessibility to viral load testing was the term ‘non-progressor’. This is relatively common in children compared to adults and includes patients who maintained age-appropriate normal CD4 counts in the absence of ART despite high levels of viraemia.^11^

Paediatric HSCs are a distinct group from adult HSCs but are exceedingly rare, and little research has been done on them. The estimated prevalence of HSCs in adults is very low, ranging between 0.18% and 0.56% of adults living with HIV.^5,6,17,18,19^ There is very little data to estimate the prevalence of paediatric HSCs but Vieira et al. has suggested that it could be as much as 5–10-fold lower than in adults.^1^ There is a female predominance in both adult and paediatric elite controllers,^1,5^ thought to be due to higher levels of immune activation that is more effective at suppressing replication in acute infection.^11^ Two of the three patients described in this case series are female. A predominance of control in people of African descent is observed in adult HSCs^4,5^ as well as some paediatric cohorts,^1^ but further research is required. In this case series, the patients are either mixed race with paternal African ancestry (Patient 1) or of African ancestry from maternal and paternal sides (Patients 2 and 3).

Differences between adult and paediatric HSCs relate to their respective immune systems. Paediatric patients are much more immunotolerant than adults, which typically prevents the development of an effective CD8+ T-cell response to HIV with consequent higher average viral loads.^11^ Mechanisms of HIV control in children are also believed to be different, as HLA class I alleles associated with control in many adult controllers are often not present in paediatric controllers.^20^ Differences in immune systems and mechanisms of control also seem to affect the time taken for paediatric and adult controllers to achieve control. Vieira et al. found in a case series that 11 paediatric controllers took a median of 6.5 years to achieve control, as opposed to adults who achieve a viral set point six weeks after infection.^1^ In adults various mechanisms of control have been proposed: specific viral and host immune factors (e.g. viral accessory gene *nef* deletion), host immune responses (CD4+ and CD8+ T-cell responses) and host genetic factors (e.g. HLA-B*5701).^21^ Recently, Vieira et al. described two distinct mechanisms leading to the loss of virological control in three paediatric TA patients.^22^ In two of the patients, an initial strong CD8+ T-cell response to an immunodominant epitope of the HIV genome initially maintained control but subsequently led to escape mutations that were not suppressed by variant-specific responses. The third patient had transient viraemic episodes that did not result in escape mutations or drop in CD4 count, but were subsequently controlled by a strong CD8+ T-cell response.^22^ This highlights the balancing act between immunotolerance and a strong immune response required to achieve and maintain control. Vieira et al. has also described different immunophenotypes in paediatric controllers and non-progressors compared to adults. Paediatric non-progressors have an initial immunotolerant environment with low levels of activation which would otherwise reduce CD4 counts, whilst paediatric elite controllers show a strong, polyfunctional CD8+ T-cell response at the time of aviraemia.^23^ The HIV reservoirs in adult elite controllers are much smaller than in those on ART, and although replication-competent provirus can be detected, it appears to be in deep latency and not transcriptionally active.^24^ However, Turk et al. recently found only replication-incompetent virus in one adult elite controller, which would negate the need for ART initiation.^25^ There are as yet no similar data in paediatric HSCs, to our knowledge.

Little is known about the natural history of HIV control in children. Adult HSCs maintain control for between 1.5 and 11.3 years.^5^ Transient viral blips with return to undetectable levels are associated with subsequent loss of control.^5^ Other factors preceding loss of control include poor T-cell responses to HIV, viral replication and ongoing evolution and markers of CD8 T-cell activation and exhaustion.^26,27^ HIV-1 control is believed to occur early after seroconversion in adults, but there is limited data on what happens during acute infection.^4,28^ Elite controllers have a lower risk of progressing to AIDS and longer maintenance of a CD4 count above 350 cells/mm^3^ compared to viraemic controllers.^4^ Hepatitis C co-infection, although a risk factor for complications (e.g. cancer, cardiovascular events and death) in adult elite controllers, was not a risk factor for loss of HIV control.^29^

Adult and paediatric HSCs present various clinical dilemmas, including confirming their HIV-positive status. Low levels of viraemia can give negative or indeterminate results on standard HIV-1 DNA/RNA and antibody tests. Patient 1, for example, had an indeterminate DNA PCR test and low levels of viraemia, requiring antibody testing and more sensitive viral testing. Confirmation of HIV-positive status was less equivocal with Patients 2 and 3. Once an HIV diagnosis has been confirmed, caution should be shown before labelling a patient with a low or undetectable viral load as an HSC. Patient 1 could have received ART via breastmilk in sufficient quantities to suppress viraemia. A similar case has been reported by Strehlau et al., where an initial HIV negative diagnosis was questioned when testing of stored samples showed the presence of proviral DNA in the child. They hypothesised that viraemia was suppressed by ART in breastmilk.^30^ Both Patients 1 and 2 could also have been receiving undisclosed ART and the only way to unequivocally prove the diagnosis as an HSC is to perform ART drug-level testing. This was done in Patient 3.

Another clinical problem is whether HSCs should initiate ART. High levels of viraemia are considered the driving force behind decline in CD4 counts and disease progression,^31,32,33^ which is not a concern in HSCs. However, high levels of viraemia are not the only concern as adult HSCs have higher levels of inflammatory markers than HIV seronegative and ART-suppressed patients.^34^ This chronic inflammatory state increases the risk of cardiovascular disease due to early atherosclerosis.^35,36^ What implications these findings in adults have for paediatric HSCs is unclear and more research is required. The prospect of paediatric HSCs maintaining control and aging into the realm of adult HSCs also presents questions. How will the stronger immune response present in adult patients impact the delicate balance of immunotolerance and increased activation present in paediatric HSCs? They may lose viral control or could have reduced life expectancy due to chronic inflammation.

Decision-making regarding ART initiation in patients who maintain control must be individualised.^37^ Factors to be considered include the following: levels of viraemia, CD4 count, CD4:CD8 ratio, disease progression, markers of heightened inflammation and T-cell activation, risk of HIV transmission, presence of replication-competent provirus and patient preference.^11,13,37,25^ For Patients 1 and 2, the decision to start ART was simple due to increasing viraemia, along with low CD4:CD8 ratios. Patient 3 required more in-depth assessment. As mentioned above, Hepatitis C co-infection has been identified as a risk factor for complications in adult elite controllers,^29^ but Patient 3’s serology was negative. Highly sensitive CRP is a marker of HIV disease progression and cardiovascular disease, independent of CD4 counts and HIV-1 RNA levels.^38,39^ Her CRP level was 0.7 mg/L, which is reassuring as a level below 1.2 mg/L is associated with slower HIV disease progression^38^ and below 3.3 mg/L with slower development of cardiovascular disease.^40^ As mentioned, the concerning factors supporting ART initiation were low CD4:CD8 ratio, more frequent viral load blips and a CD4 percentage below 40%. Bansal et al. had shown that a CD4 percentage below 40% in adult HSCs was associated with increased markers of T-cell and monocyte activation.^41^ Patient 3 agreed to initiate ART and has since maintained viral suppression.

## Conclusion

Paediatric HSCs are a very important population to study and advance the HIV ‘Cure Agenda’. Mechanisms by which they can spontaneously suppress HIV viral replication may hold the key to future novel therapeutic approaches and curative measures, and further research is urgently needed. Clinical dilemmas involved in their care include appropriate diagnosis of HIV, confirmation of an HSC diagnosis and whether ART initiation is appropriate. Paediatric HSCs therefore require specialised and individualised care, despite comprising a small section of the paediatric population living with HIV.
